# How children in Sweden accessed and perceived information during the first phase of the Covid-19 pandemic

**DOI:** 10.1177/14034948211051884

**Published:** 2021-11-05

**Authors:** Lise-Lott Rydström, Charlotte Ångström-Brännström, Lucy Blake, Lucy Brayl, Bernie Carter, Maria Forsner, Janet Matsson, Stefan Nilsson, Margaretha Jenholt Nolbris, Jennifer Kirton, Inger Kull, Joanne Protheroe, Anna-Clara Rullander, Holly Saron, Anna Lindholm Olinder

**Affiliations:** 1 Department of Neurobiology, Care Sciences and Society Karolinska Institutet, Sweden; 2 Department of Nursing, Umeå University, Sweden; 3 Department of Women’s and Children’s Health, Uppsala University, Sweden; 4 Department of Social Science, University of the West of England (UWE); 5 Faculty of Health, Social Care and Medicine, Edge Hill University, Ormskirk, UK; 6 Department of Biosciences and Nutrition, Karolinska Institutet, Sweden; 7 The Swedish Red Cross University College, Department of Health Sciense, Sweden; 8 Department of Learning, Informatics, Management and Ethics (LIME), Karolinska Institutet, Sweden; 9 University of Gothenburg Centre for Person-centred Care’, University of Gothenburg, Sweden; 10 University of Gothenburg Sahlgrenska Akademi, Health and Care Sciences and The Queen Silvia Children’s Hospital, Sweden; 11 Department of Clinical Science and Education, Södersjukhuset, Karolinska Institutet, Sweden; 12 Sachs’ Children and Youth Hospital, Södersjukhuset, Sweden; 13 FRCGP Keele Medical School, Keele University, UK

**Keywords:** Children 7-12 years, survey, drawings, information, Covid-19, outbreak phase, health literacy

## Abstract

**Aim:**

To describe how children in Sweden accessed and perceived information about SARS-CoV2 and Covid-19 during the first phase of the outbreak.

**Methods:**

This study is a substudy of an international cross-sectional online mixed methods survey examining elements of children’s health literacy in relation to Covid-19. The survey included multiple-choice questions, open-ended questions and drawings and collected information from 50 Swedish children (7–12 years). Data were analysed concurrently on a descriptive level using statistics and content analysis. Quantitative and qualitative data, including the drawings, were considered equally important and resulted in six categories, illuminating how children accessed and perceived information about the pandemic.

**Results:**

The survey showed that children accessed information mainly from school but also from TV. They preferred information from reliable sources. Children reported the information they accessed as easy to understand and it prompted them to ask new questions. They reported they knew a lot about the pandemic, for example, the potential danger to themselves and others and how to act to protect themselves and others. They perceived the pandemic as an intrusion on their lives.

**Conclusions:**

**This study indicates that Swedish children between 7 and 12 years old were well informed about SARS-CoV2 and Covid-19 during the first phase of the pandemic. School was shown to be an important source of information. The children could explain how to act to protect themselves and others from becoming infected by the virus.**

## Introduction

In the beginning of 2020 news about a previously unknown viral disease reached the world. The situation in society which resulted from the Covid-19 pandemic was new and has been described as frightening for both children and adults [1].

Covid-19 was classified as a socially dangerous disease in Sweden in February 2020, and in the middle of March people were recommended to stay at home if they had symptoms or had passed the age of 70 years. Senior high schools and universities were closed, but schools with pupils up to 15/16 years of age remained open [2]. There was no general lockdown of society [3].

The pandemic has created changes in daily life [1], socioemotional and financial stress for many families [4], and mental health challenges [5]. Although children seem to experience less severe illness and symptoms from Covid-19 than adults [6], the pandemic has led to consequences such as negative changes in wellbeing and health [1], limited connection with friends, reduced physical activities [5, 7] and stress and fear of the death of relatives [5].

In addition to concerns about their family’s situation, parents have a responsibility to ensure that their children have information and knowledge about a situation and the recommendations to follow to mitigate the spread [2, 5, 8]. Also, school professionals can address public information about the pandemic in class [9], to ensure children’s rights to access health education [10]. Health literacy (HL) is a prerequisite for a person to have a fair opportunity to attain full health potential. It refers to the ability of individuals to gain access, understand and use information in ways that promote and maintain good health for themselves, their families and their communities [11]. Paakkari et al. [12] claim that health behaviour is established during childhood and adolescence, and that education has a crucial role in the development of HL. Five core components of HL in adolescence are identified: theoretical knowledge, practical knowledge, critical thinking, self-awareness, and citizenship [12]. However, children can find it difficult to interpret health information told to them or viewed online or on TV [13]. Overall, HL has been researched more in adolescents than in younger children [14, 15], and mostly with respect to related measures such as health knowledge [15], which might explain the reason that HL as defined above presupposes autonomous decision-making competence not developed in younger children [14]. Elements of HL might be present in younger children, namely, theoretical knowledge, as according to Paakkari et al. [16] this includes lower levels of thinking skills such as remembering received information and practical knowledge. Velardo and Drummond emphasise the need for examining children’s perspectives to expand our knowledge about the ways in which children receive, make sense of health messages and navigate contemporary health information across developmental stages, especially in middle childhood and pre-adolescence [14].

It is of interest to investigate HL in Swedish children related to the pandemic during the first phase of the pandemic, as HL in children is poorly investigated, the Covid-19 pandemic has led to an abundance of public health information, and because Sweden did not close down to mitigate the spread of the virus.

Considering the children’s developmental stage and with the desire to capture the moment, the study focusses on elements of HL, rather than in the multidimensional meaning, namely how they access and perceive information about the virus, the disease and how to act during the pandemic.

### Aim

To describe how children in Sweden accessed and perceived information about SARS-CoV2 and Covid-19 during the first phase of the outbreak.

## Methods

### Design

This study is reporting on a subsample of an international cross-sectional online survey examining elements of children’s HL, such as information sources children accessed, children’s information preferences, their perceived understanding of and their reported information needs in relation to Covid-19 and SARS-CoV2 [8]. In this paper we report data from the Swedish children.

To capture children’s perceptions and experiences a convergent parallel mixed methods design was applied, inspired by Creswell and Plano Clark [17], in which quantitative and qualitative data, including drawings, were collected simultaneously and considered equally important.

### Study procedure

The online survey was open for Swedish participants from 22 May to 1 June 2020. A pamphlet providing information about the study and a link to the survey were distributed online (e.g. school websites, Facebook, Instagram and interest groups) directed to the children themselves as well as parents of children in the current age group and expanded through snowballing. Children and caregivers were informed about the purpose and execution of the study, about the primary investigator in the UK and in Sweden, that participation was voluntary, anonymous, and that a submitted survey would be perceived as their assent to take part in the study. The caregivers provided consent for their child to participate in the study by ticking a statement before their child could respond to the survey. The study was reviewed by the Swedish Ethical Review Authority (DNR2020-02351).

### Measurements

The online survey was translated from English to Swedish and back translated by two different professional interpreters. The back translation was checked by the team from the UK and then the Swedish version was discussed within the Swedish team. Thereafter the survey was pilot-tested among five children, in the same intended age group as participants. The survey included two demographic questions about age and domicile, four multiple-choice questions focused on information and knowledge about SARS-CoV2 and Covid-19, five open-ended questions about the main sources of information, how children wanted information to be provided, three things they knew, three things they would like to know and three words they thought about when thinking of things related to SARS-CoV2 and Covid-19 [8].

In addition, children were invited to draw and label a picture and attach it to the survey, answering the question ‘Can you provide a drawing explaining how to act during the corona pandemic?’ Asking children for drawings aimed to enable access to children’s understandings and thoughts related to the pandemic and to generate rich data [18], because art expression allows them to share their thoughts and to provide an understanding of their worlds [19, 20].

#### Analyses

The quantitative and qualitative data were analysed separately in parallel [17].

##### Statistical analysis

Descriptive statistics (percentages and frequencies) counted with IBM SPSS Statistics, version 27 (IBM Corp., Armonk, NY, USA) were used to describe the population, children’s knowledge and how they got information about SARS-CoV2 and Covid-19.

##### Qualitative analysis

The children’s responses to open-ended questions were subjected to content analysis with an inductive approach. The text was read through several times, an open coding was performed, followed by grouping into six preliminary subcategories [21]. As the data consisted of anonymous open text answers, the analysis was limited to manifest content not to over-interpret the children’s statements [22].

Twenty drawings were produced, 13 monochrome and seven in colour, with explanatory text in all except three. The drawings were sorted and described without any further interpretation, given that children’s drawings are individual [19] and ought to be considered within the larger context of their developmental, emotional, social and cultural experience [20].

##### Mixed analysis

The findings from the quantitative and qualitative data, including the drawings, were then compared, discussed and found to strengthen each other; that is, convergent [17], leading to six integrated categories in which both quantitative and qualitative data illuminate how children accessed respectively perceived information.

Quotations from the open-ended responses and drawings illustrate the results.

To ensure an accurate translation of children’s Swedish words into English, translation was performed by two bilingual children (12 and 14 years old) in discussion with the last author.

## Results

The 50 children who participated were 7–12 years old (mean 9.7 ± 1.8) and lived across the whole of Sweden. Sixteen (32%) were from the north and 14 (28%) were from the western side of Sweden, and the rest of the participants were from different regions across the whole country. Nearly all children (94%, *n*=47) reported school attendance.

### Accessing information

#### Receiving information

According to their responses the participants had received information about SARS-CoV2 and Covid-19. The main sources of information reported were from the school 90% (*n*=45) and from *Lilla Aktuellt* (news for children) 78% (*n*=25). Other sources of information were the radio (34%, *n*=17), parents (26%, *n*=13), friends (26%, *n*=13) and social media (26%, *n*=13). Likewise, in the open-ended questions, school, parents, people with knowledge, and media, such as news for children and radio, were mentioned as sources of information.

Talking about the pandemic was found to be important (40%, *n*=20) and interesting (34%, *n*=17). However, 30% (*n*=15) found it boring, or did not talk about it. In the open-ended responses, it was mentioned that information about the virus could make them feel ill at ease: ‘Ideally I would not like to know anything about Corona. . ..’

#### Preferring reliable sources of information

Texts and drawings showed that children prioritised reliable sources of information, and relatives and friends were mentioned as such sources.

**Figure 1.**
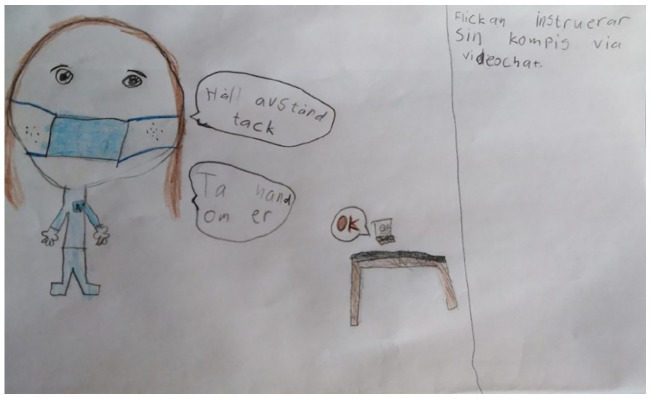
The girl instructs her friend through video chat, and the text says, ‘Keep distance, please’ and ‘Take care of yourselves’.

Information through reliable sources was reported as more calming than getting new information every day through a newscaster on TV (Figure 1). ‘I would like information from my mum because she works with Covid-19 and a lot of other things in healthcare, so I trust that she knows her stuff.’

Likewise, personal information from an expert was preferred. ‘That somebody comes and knocks [on the door] – and tells me.’

#### Wanting to know more

There was a desire to know more about the virus, regarding, for example, the appearance of the virus, how and why it arose, why it exists and why it is called Covid-19. ‘What does it [the virus] look like’?

**Figure 2.**
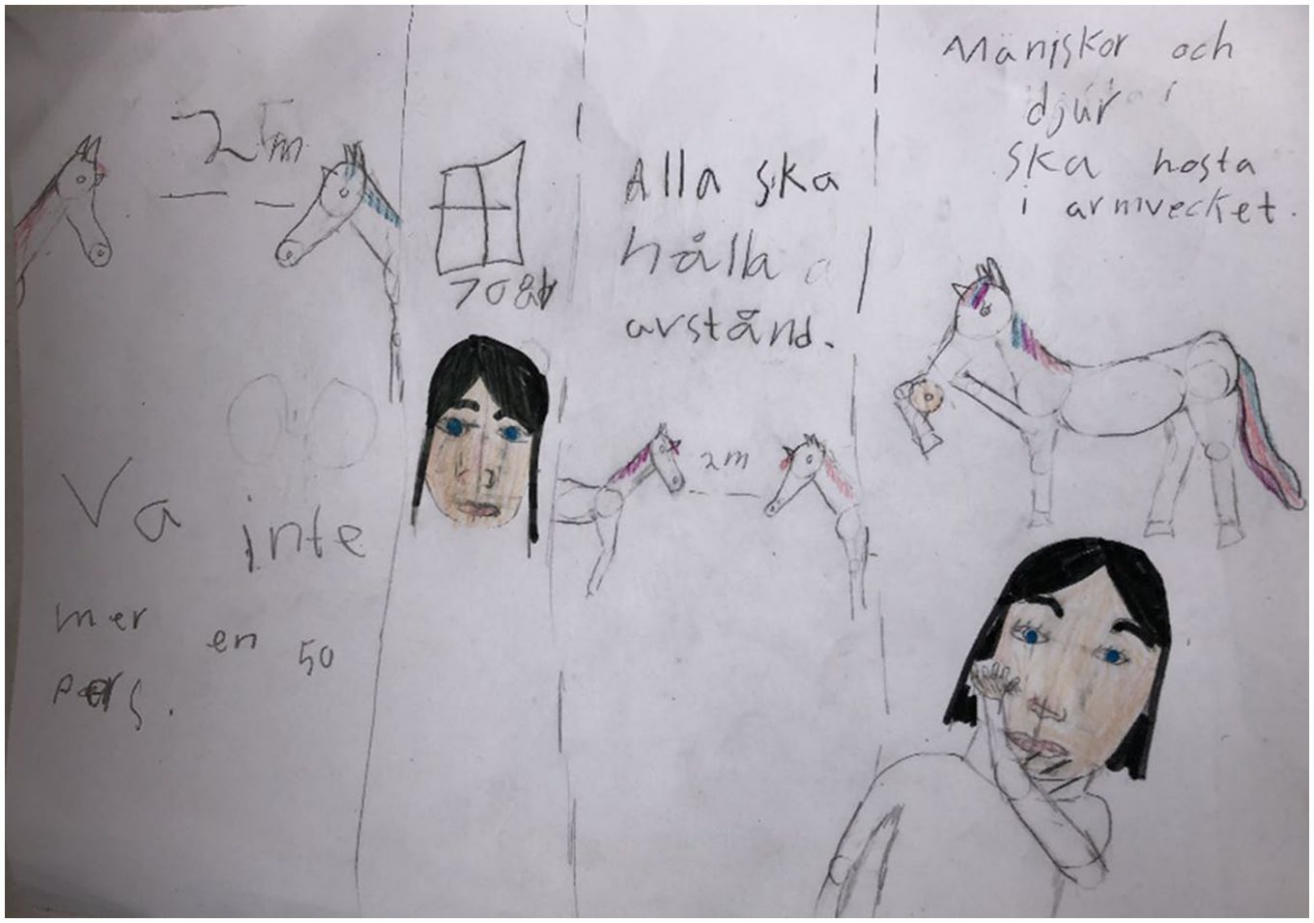
The text says, ‘2 meters distance’, ‘Do not gather in groups with more than 50 people’, ‘[People over] 70 years [should be isolated]’, ‘Everybody must keep distance’, and ‘People and animals must cough into the arm’.

Children raised thoughts and questions about why children do not get as sick as older people do, and also identified questions regarding if animals could be infected (Figure 2). They also wanted to know if they had had the virus, if infants could get sick, and if it is possible to prevent the virus from coming back. ‘How to stop it?’

Common questions were also raised about when the pandemic would end, when a vaccine would be available, if there would be any cure, how many people have died from the virus, and the number of deaths there will be before the pandemic is over. ‘How many will die from Corona?’

The written comments also questioned some behaviour children had seen during the pandemic such as crowding and stocking food.

### Perceiving information

#### Knowing a lot

The multiple-choice questions indicated that most of the children, 80% (*n*=40), reported that they found the information about Covid-19 they had accessed to be very easy/easy to understand, 20% (*n*=10) did not know if it was easy or difficult, or found it difficult. Sixty-six per cent (*n*=33) of the children thought that they knew a lot/quite a lot about Covid-19, 34% (*n*=17) thought that they knew a little/not so much or did not know how much they knew.

In comments children’s knowledge about the virus were demonstrated, for example, ‘it’s green’, ‘it started in China’ and ‘it came from bats’.

They demonstrated knowing about how the disease manifests itself, because the most common words they reported in relation to Covid-19 was ‘cough’ and ‘breathing problems’. Their responses also indicated that they were aware how the virus was transmitted: ‘It infects with droplet’ and of the possibility of carrying the virus without any symptoms or with just mild symptoms. The drawings reflected the recommendations from the Public Health Agency of Sweden.

#### Potentially dangerous but not to children

Covid-19 was described as a lethal disease. ‘You can die from the virus if you are unlucky’. Children reported that people all over the world were dying from it and that different countries were affected in different ways. They told that it was mainly older people who died and should be isolated, and that the virus was of less or no danger to themselves or their parents if they were healthy and had no other underlying diseases. ‘It is more dangerous for the elders and those with less resilience’.

#### Intruding on life

The children’s drawings and text indicated the massive impact the pandemic had had on their lives; wherever they went, people were talking about Covid-19. ‘That again. . .. It’s always on the news’. The fact that you must maintain social distancing affected their daily life and life became limited. They were unable to see their grandparents, and to travel in the country or abroad. ‘You can’t go to Italy now’. Even the school graduations were cancelled. They described how their summer plans had been disrupted and spoilt due to the pandemic and the associated restrictions.

On the other hand, the participants were aware that children’s restrictions in other countries were more rigid and that children were not allowed to go to school.

#### Protecting yourself and others

**Figure 3.**
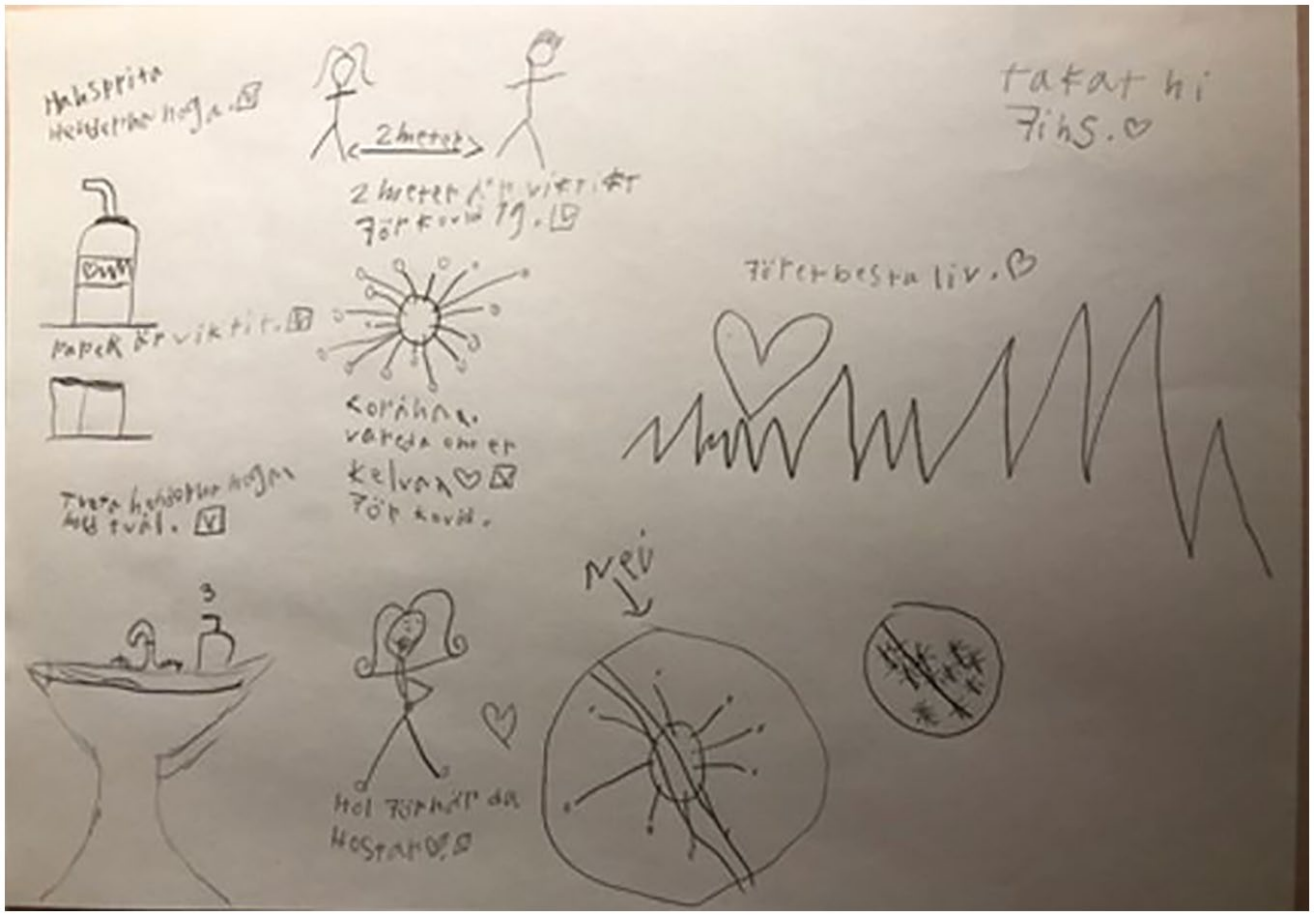
The text says: ‘Use hand sanitizer carefully’, ‘Paper towels are important’, ‘Wash your hands thoroughly with soap’, ‘2 meter distance is important for Covid-19 ‘, Care for yourself’, ‘Cover your mouth when you cough’, ‘For the best life’, ‘Thank you for being here’.

**Figure 4.**
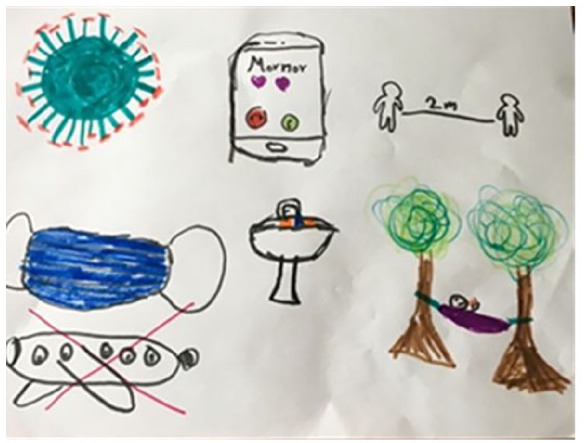
The text on the cell phone says ‘Grandmother’.

**Figure 5.**
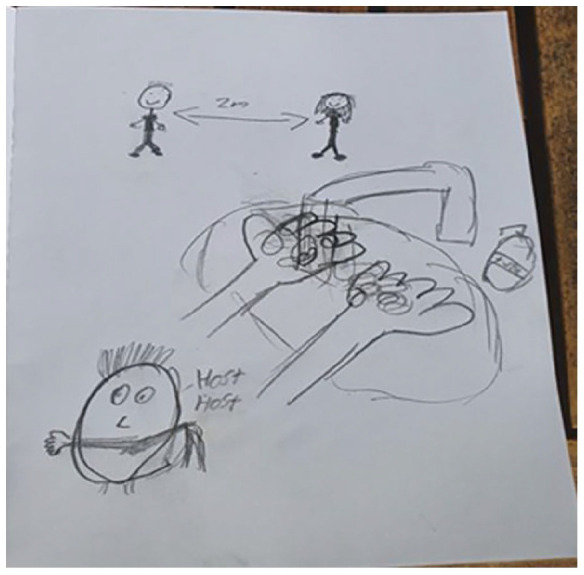
The text says ‘2 meter (distance)’, ‘soap’ and ‘Cough, cough’.

The drawings exhibit how the children perceive ways of protecting others and themselves (Figures 3, 4 and 5). That you should wash your hands often, use rubbing alcohol, and not hug or shake hands and keep distance. ‘Keep at least two metres distance from each other’.

The children’s drawings depicted people and/or stick figures in different contexts inside and outside. Children depicted activities that should be avoided, like gathering in groups, and activities that should be encouraged, like playing outside. Eight drawings depicted people and animals wearing face masks (e.g. Figure 1).

Other things children described were to stay home if you felt sick and quarantine to avoid risking infecting others. Vaccines were mentioned as a way to protect others and themselves.

## Discussion

The findings of this study gave a glimpse into how Swedish children, aged 7–12 years, accessed and perceived information about SARS-CoV2 and Covid-19 during the first phase of the pandemic. Compared to other countries, where parents were the main source of information [8], it was clear that school significantly contributed to the Swedish children’s knowledge about Covid-19. Paakkari et al. suggest that HL is improved by being a compulsory subject in school [12]. According to the Swedish curriculum for compulsory school they must support pupils to gain knowledge of and understanding of the importance of their own lifestyle for health, the environment and society [9].

In Sweden, schools up to 9th grade (15/16 years of age) were open during the time of the survey [2], and nearly all the participating children had been able to go to school, thus it was possible for children to get their information from school. The participating children appreciated personal information from relatives, friends and knowledgeable persons. Children in this age group need to reflect on their knowledge together with others, to develop higher-level thinking skills in HL [16].

The children accessed information from different media, including television and the internet. They expressed that they wanted information from reliable sources, which highlight the importance of children getting truthful information in age-appropriate language [13] such as ‘*Lilla Aktuellt*’; specifically designed for children and often used in schools. One for HL-related competence is Media and Information Literacy (MIL), including literacy in media, news, the internet, computers, games, advertising, cinema and television [23]. It can be argued that children of today possess these notions, perhaps even to a greater extent than adults. However, higher-level cognitive skills such as critically evaluating media content [16] are due to developmental factors not supposed to be fully achieved in children of these ages. Therefore, they need clear, honest answers in response to their questions about the pandemic. As emphasised in the Swedish curriculum, it’s good to talk with children about media reports and help them develop a critical understanding of these sources of information [9].

The results indicated that the Swedish children found information about the pandemic easy to understand, which was also the case for children from the UK, Spain, Canada, Australia and Brazil [8]. This is in line with Fretian et al. who found that 82% of 9 and 10-year-olds in Germany found it easy to handle health information [15]. However, their conclusion recognised that the majority of this age group are healthy and do not need to orientate themselves to healthcare and health information [15]. Arguably, the context of the pandemic is similar because most children have not been found to be seriously affected by Covid-19.

An interesting finding in this study was that in eight of the 20 drawings people and animals were depicted using face masks. The question is who told the children about face masks; had they seen people in other countries wearing them on newscasts? At the time of the data collection, face masks were only recommended for healthcare personnel in Sweden [3].

Children have the right to health education and to be engage in decisions about their own situation [10], and HL is crucial for making this possible. This study illuminates how schools are a key source of health information to children during a pandemic. UNICEF has called on the world’s decision-makers to pay attention to children’s situations when taking action against the spread of SARS-CoV2 [24], and closing schools must therefore be weighed against the benefits of schools keeping children informed about the situation.

### Limitations

This study was limited to the reports from 50 children who had been able to access and respond to the survey during a short period of time, the last 10 days in May 2020. The children were reached through social media and the survey was anonymous and kept simple with just a few questions, leaving us with restricted information about the participants and how they were recruited. Despite these limitations, the results can yield a snapshot of how children reported accessing and perceiving information about SARS-CoV2 and Covid-19.

The desire to catch this specific phase of the pandemic prevented a more in-depth investigation. Perhaps face-to-face interviews might have been preferable. On the other hand, children of this age are supposed in writing to be able to express their thoughts and perceptions in direct and structured questions [25] as well as open-ended questions [22, 25]. The findings in this mixed methods study was reinforced by combining multi-choice and open-ended questions with drawings as a child-centred, developmentally appropriate method to help children share their opinions and experiences [20]. However, as it was not possible to follow up what the children meant with their statements or drawings, caution has been applied not to overinterpret data. Analysis was limited to describing the content [22]. Quotes and drawings are included to provide transparency.

## Conclusions

This study indicates that Swedish children were well informed about SARS-CoV2 and Covid-19 during the first phase of the pandemic. Children reported that TV and school were important sources of information, and children were able to explain how to act to protect themselves and others from being infected by the virus.
